# 704 Ceftazidime / Avibactam Efficacy for Treating Carbapenem-resistant Infections in Critically Ill Patients with Thermal Injuries

**DOI:** 10.1093/jbcr/irac012.258

**Published:** 2022-03-23

**Authors:** David M Hill, Kelsey Martin, Faisal Arif, Ibrahim Sultan-Ali, Sai R Velamuri

**Affiliations:** Firefighters' Burn Center, Memphis, Tennessee; Firefighter's Burn Center, Memphis, Tennessee; Firefighter's Burn Center, Memphis, Tennessee; Firefighter's Burn Center, Memphis, Tennessee; Firefighter's Burn Center, Memphis, Tennessee

## Abstract

**Introduction:**

Rising antimicrobial resistance is a pressing public health concern. Emergence of carbapenem-resistant organisms has led to increased use of novel antibiotics, such as ceftazidime/avibactam (CZ/AV). However, recent studies have shown increasing treatment failures and resistance rates associated with ceftazidime/avibactam use. The efficacy of CZ/AV has not been studied in patients with thermal injuries, where pharmacokinetic derangements are common and longer lengths of stay augment risk of requiring several antimicrobial courses, leading to higher resistance rates. The objective of this study was to evaluate the outcomes of patients with thermal injuries including clinical success, the frequency of adverse effects, and emergence of resistance.

**Methods:**

The design was a retrospective chart review. Patients were included if admitted with thermal injuries and receiving at least 48 hours of CZ/AV between Jan. 1, 2017 and July 2020. Demographics and treatment data were reported using descriptive statistics. Treatment success, description of treatment failures, and adverse events were described. Logistic regression was used to control and analyze failures.

**Results:**

Fifteen patients with 17 courses evaluated. Most were male (87 %) and African American (53 %). The mean age and weight was 47.7 ±13.6 and 96.3 ± 29.4. Seventy-three percent had a flame injury. Mean TBSA was 34 ± 18.7. Twenty percent had an inhalation injury and 80 % a significant substance use history. Clinical success occurred in 65% (11/17) although 29% died. E. cloacae (88%) was the most common treated pathogen, but 81% were polymicrobial. The most common sources were wounds (29%), blood (29%), and lungs (26%). Median days until CZ/AV initiation was 32 (14,76). CZ/AV was dosed at 2.5 g every 8 hours in all courses. Median treatment duration was 12 days (9,14). Fifty-three percent received CVVH with a mean delivered dose of 47.6 ± 9.5 ml/kg/hr. Resistance developed in 19% (3/17) of courses, but follow up sensitivities were rarely available. Logistic regression did not reveal any variables significantly associated with failure. There were no adverse events attributed to CA/AV

**Conclusions:**

Although lower than desired, clinical success rates in this sample were similar to other reported populations treated with CZ/AV. However, the emergence of resistance occurred more frequently, and was likely underreported in this sample. HVHF did not contribute to failure, but CZ/AV was aggressively dosed in this cohort.

